# Root morphological and physiological traits are committed to the phosphorus acquisition of the desert plants in phosphorus-deficient soils

**DOI:** 10.1186/s12870-023-04178-y

**Published:** 2023-04-10

**Authors:** Yanju Gao, Zhihao Zhang, Fanjiang Zeng, Xingyu Ma

**Affiliations:** 1 Xinjiang Key Laboratory of Desert Plant Roots Ecology and Vegetation Restoration, Xinjiang Institute of Ecology and Geography, Chinese Academy of Sciences, Urumqi, 830011 China; 2 State Key Laboratory of Desert and Oasis Ecology, Xinjiang Institute of Ecology and Geography, Chinese Academy of Sciences, Urumqi, 830011 China; 3 Cele National Station of Observation and Research for Desert-Grassland Ecosystems, Cele, 848300 China; 4 University of Chinese Academy of Sciences, Beijing, 100049 China

**Keywords:** Root traits, Desert ecosystem, Phosphatase activity, Carboxylates, P limitation, P acquisition

## Abstract

**Background:**

Phosphorus (P) deficiency in desert ecosystems is widespread. Generally, desert species may allocate an enormous proportion of photosynthetic carbon to their root systems to adjust their P-acquisition strategies. However, root P-acquisition strategies of deep-rooted desert species and the coordination response of root traits at different growth stages to differing soil P availability remains unclear. In this study, a two-year pot experiment was performed with four soil P-supply treatments (0, 0.9, 2.8, and 4.7 mg P kg^–1^ y^–1^ for the control, low-, intermediate-, and high-P supply, respectively). Root morphological and physiological traits of one- and two-year-old *Alhagi sparsifolia* seedlings were measured.

**Results:**

For two-year-old seedlings, control or low-P supply significantly increased their leaf Mn concentration, coarse and fine roots’ specific root length (SRL), specific root surface area (SRSA), and acid phosphatase activity (APase), but SRL and SRSA of one-year-old seedlings were higher under intermediate-P supply treatment. Root morphological traits were closely correlated with root APase activity and leaf Mn concentration. One-year-old seedlings had higher root APase activity, leaf Mn concentration, and root tissue density (RTD), but lower SRL and SRSA. Two-year-old seedlings had higher root APase activity, leaf Mn concentration, SRL and SRSA, but a lower RTD. Root APase activity was significantly positively correlated with the leaf Mn concentration, regardless of coarse or fine roots. Furthermore, root P concentrations of coarse and fine roots were driven by different root traits, with root biomass and carboxylates secretion particularly crucial root traits for the root P-acquisition of one- and two-year-old seedlings.

**Conclusions:**

Variation of root traits at different growth stages are coordinated with root P concentrations, indicating a trade-off between root traits and P-acquisition strategies. *Alhagi sparsifolia* developed two P-activation strategies, increasing P-mobilizing phosphatase activity and carboxylates secretion, to acclimate P-impoverished in soil. The adaptive variation of root traits at different growth stages and diversified P-activation strategies are conducive to maintaining the desert ecosystem productivity.

**Supplementary Information:**

The online version contains supplementary material available at 10.1186/s12870-023-04178-y.

## Introduction

Desert ecosystems feature low productivity and are increasingly found to be characterized by phosphorus (P)-deficient soil, considered to be a key limitation to desert species’ survival [1, 2]. P actively participates in various physiological and biochemical processes in plants, including protein synthesis, enzyme activation, lipid membranes, photosynthesis, and respiration [3–6]. Accordingly, how desert species adapt to their P-deficient environment is a potential ecological problem when preserving desert ecosystem functioning [7, 8].

Generally, plants respond to the P-deficient soil mainly by improving their P-use efficiency aboveground or increasing their P-acquisition rate belowground [9, 10]. Plants efficiently utilize aboveground P by it rational distribution (i.e., its priority allocation to young and vigorous growing parts), re-absorption in leaves before their senescence, and redistribution of internal P [3, 11]. For instance, Gao et al. [7, 12] investigated the desert species growing in different soil P-availability sites under natural conditions, finding that *Alhagi sparsifolia* and *Tamarix ramosissima* were mainly limited by P, whose strategies to adapt to local P limitation included reducing their P requirements aboveground and improving the flexible allocation ability of foliar-P fractions with different functions. Belowground, however, given that plant roots constitute an interactive interface with the soil environment, they play a crucial role in obtaining nutrient resources, especially those in limited supply [13, 14]. A global mata-analysis showed that up to 56% of photosynthetic productivity can be allocated belowground to plant roots in desert ecosystems [15]. In addition, up to 30% of photosynthetic productivity may be devoted to the fine roots (diameter < 2 mm) having strong uptake functions for nutrients and water [16]. Desert plants are distinguished by the most photosynthetic productivity allocated to roots, to adapt to harsh arid and soil nutrient-deficient conditions [17]. Consequently, root traits of desert species are considered vital drivers of crucial processes for the functional components of desert ecosystems, including plant community dynamics, soil carbon accumulation, soil nutrient cycling, and plant nutrient requirements [18]. Nevertheless, the integrated root traits of deep-rooted perennial plants growing in P-deficient conditions are currently poorly understood due to these roots reaching deep into groundwater in the natural ecosystem.

P is generally easily fixed in the soil by some metal ions (e.g. Fe^2+^, Al^3+^ and Ca^2+^) and organic matter, which makes P availability very poor in soil [19, 20]. Thus, plant roots must actively move towards P [21]. Previous studies found that thinner and longer roots are conducive to exploring a greater soil volume in patches of P [6, 22], and a larger surface area (SA) of roots contributes to P acquisition by increasing the total absorption interface area [23]. Therefore, fine roots are considered a key organs for plants’ uptake of nutrients and water, especially for some perennial xerophytes whose coarse roots because highly lignified [17]. In addition, previous studies have explored the potential associations between P availability in soils and several root traits, such as specific root length (SRL: root length per gram root biomass), specific root surface area (SRSA: root surface area per gram root biomass), root tissue density (RTD: root biomass per unit root volume), and root biomass [24]. For instance, a P treatment significantly improved the total root length (RL) and root biomass of *Lolium perenne* and 6-month-old *Populus cathayana*, but decreased their SRL, vis-à-vis the control treatment [6, 25].

Root physiological traits tend to be more closely associated with the availability of soil nutrients [14, 22]. For instance, when faced with P deficiency in the soil, plant roots will release large amounts of P-mobilizing exudates, such as phosphatase (intracellular and extracellular), carboxylates, and protons, to remobilize, recycle, and hydrolyze the organic phosphate (Po) and occluded inorganic phosphate (Pi) in soils [26–28]. Among them, phosphatase strongly hydrolyzes soil Po, while carboxylates can promote the dissolution of insoluble P-containing compounds by ionizing H^+^ and lowering the soil pH [29]. Previous studies have indicated that some plants (e.g., Myrtaceae) prefer to release phosphatase [24], while others (e.g., Proteaceae and Fabaceae) prefer to release carboxylates to activate soil P [10]. Therefore, P-mobilizing phosphatase and carboxylates in the rhizosphere are considered crucial factors in the root response to P-deficient soils [30]. Nevertheless, there are many difficulties and inaccuracies that arise when extracting carboxylates from the soil [29]. Surprisingly, a robust linear relationship between the leaf Mn concentration and rhizosphere carboxylates’ concentration was found, which is why the leaf Mn concentration of a plant is advocated as suitable proxy for the concentration of its rhizosphere carboxylates [30, 31]. Furthermore, differential correlations between root traits have been reported in different soil P availability environments [32]. For instance, the RTD of 16-month-old *Syzygium castaneum* was greater under a high-P treatment, whereas SRSA and root acid phosphatase (APase) activity were greater under low-P treatment [24]. Consequently, the coordination between root morphological traits, carboxylate exudation, and phosphatase activity at different plant growth stages may enable roots to explore soil and efficiently mobilize Po and Pi.

In the Taklimakan Desert, given the low groundwater table there, the vegetation at its southern edge junction is dominated by deep-rooted perennial plants [33]. Because their root systems directly reach downward to several meters, or sometimes even more than 10 m, to uptake groundwater, studying them in the natural ecosystem poses a formidable challenge. Therefore, research on plant functional traits in this desert region has chiefly focused on easily determined aboveground traits [7, 12, 34]. The perennial *Alhagi sparsifolia* is a typical dominant species being widely distributed across the oasis landscape and to the front of the desert–oasis transition zone [1]. Not only can *Alhagi sparsifolia* be used for wind-sheltering and sand-fixation purposes, but also as an excellent resource of forage grass, hence, its research has crucial ecological value and economic implications [17, 33]. In this study, we conducted a pot experiment and selected one-year-old and two-year-old deep-rooted *Alhagi sparsifolia* seedlings, to investigate the root P-acquisition strategies and the coordination response of root traits at different growth stages under different P supply treatments (control, low-, intermediate- and high-P). The following hypotheses were tested: (i) as a typical Fabaceae, the root system of *Alhagi sparsifolia* mainly activates soil P by releasing carboxylates rather than phosphatase; (ii) the coordination among root morphological traits and root APase activity and leaf Mn concentration at different growth stages is devoted to root P-acquisition in P-deficient soils.

## Materials and methods

### Study area description

The study was conducted in an open-air artificial ecological research experimental field (37°00′57′′N, 80°43′45′′E, 1353 m elevation) located at the transition zone between the Cele oasis and desert on the southern edge of the Taklimakan Desert [17]. Here, is a warm temperate desert climate prevails, with a mean annual temperature of 15.85℃ and precipitation of 42.62 mm (hyper-arid), where the potential annual evaporation is 2700 mm [35]. The vegetation is dominated by perennial phreatophytes, such as *Alhagi sparsifolia* Shap. (Fabaceae), *Calligonum caput-medusae* Schrenk. (Polygonaceae), and *Tamarix ramosissima* Lcdcb. (Tamaricaceae), etc.

### Experimental design

A pot experiment was conducted from 2020 to 2021. Plastic pots (diameter: 60 cm, height: 100 cm) with wheels were filled with 300 kg of homogenized topsoil taken from the study area. The test soil was collected from the natural desert ecosystem (0–30 cm soil layer), air-dried for 2 days, and then sieved through a 0.5-cm mesh screen to remove large particulate matter. Collected soil was classified as sandy soil with low concentration of soil organic matter. The physiochemical properties and soil P fractions can be found in Supporting Information Tables S1 and S2.

Four soil P treatments consisting of a control (0 mg P kg^–1^ y^–1^), low-P (0.9 mg P kg^–1^ y^–1^), intermediate-P (2.8 mg P kg^–1^ y^–1^), and high-P (4.7 mg P kg^–1^ y^–1^) supply were imposed by adding 0 g, 1.39 g, 4.18 g, and 6.97 g NH_4_H_2_PO_4_ (P 27%, N 12%), respectively. Each treatment was replicated 12 times, for a total of 96 pots in the experiment (2 seedling age groups × 4 treatments × 12 pots). In addition, in order to balance the N difference caused by different P supply treatments, we added 3.21 g, 2.57 g, and 1.29 g CO(NH_2_)_2_ (N 46%) for the control, low-P, and intermediate-P treatment each pot per year, respectively, based on the N brought by the high-P treatment. In April 2020, about five seeds of *Alhagi sparsifolia* were sown in each pot after watering the soil to its maximum field capacity (ca. 18%). The pots were arranged in a completely randomized design that was re-randomized every weekly. After one month of growth, the seedlings of *Alhagi sparsifolia* with 4–6 leaves were thinned to one healthy and uniform seedling in each pot. Then, the P source was applied per pot with water once in May 2020 and again in May 2021, based on the four treatments (P supply levels). Each pot received ca. 1.5 L of water every five days in April, May, September, and October (due to low evaporation), but 3 L of water every three days in June, July, and August (due to high evaporation) to keep the *Alhagi sparsifolia* seedlings alive.

### Samples’ collection

In August 2020 and 2021 (peak growth season), the samples of coarse root (> 2 mm diameter), fine root (< 2 mm diameter), leaf and stem as well as rhizosphere soil, were collected. We selected 12 pots per treatment and then carefully sawed the pot along its height. Among them, six pots were used to obtain the entire roots, these placed in a 2-L barrel for their root image acquisition. After collecting the leaf and stem samples, the root systems of the other six pots were divided into coarse and fine roots with scissors, washed and put into resealable bags, and immediately placed inside a 4℃-refrigerator to determine root APase activity. Next, part of the fine roots with loose adhering soil was cut off, placed into a 50-mL centrifuge tube, and rhizosphere soil then obtained by centrifugation (at 4000 r min^–1^, for 15 min) for analysis of soil P pool. Finally, the root samples (coarse and fine roots) of each pot were collected, cleaned, and dried at 75℃ for 48 h, and their root biomass and P concentration determined. To distinguish between coarse and fine root material, a digital caliper was used [36, 37]. Due to the large morphological discrepancy between the coarse and fine roots of *Alhagi sparsifolia*, these are more easily differentiated. Briefly, multiple root diameters were first measured using a digital caliper, and roots with diameters > and < 2-mm were recorded. Then, the recorded root samples were used as a preliminary criterion for judging the grades of coarse and fine roots to speed up the sorting process. Finally, the digital calipers were used again to measure the diameter of a few roots whose size class was difficult to judge by eye.

### Root morphological traits

After carefully washing the roots with deionized water and removing any plant litter attached to them, integrated root samples of *Alhagi sparsifolia* were imaged on the Expression 1600 Pro scanner (Expression, Model EU-35; Epson, Tokyo, Japan). The root volume (RV, cm^3^), root length (RL, cm), and surface area (SA, cm^2^) of coarse and fine roots were analyzed using Root System Analyzer software (WinRhizo Pro 2004b software, v.5.0, Regent Instruments Inc., Quebec, QC, Canada). Then, the respective biomass of coarse and fine roots was obtained by weighing the samples after oven-drying them (at 75℃, for 48 h), according to which their specific root length (SRL, cm mg^− 1^), specific root surface area (SRSA, cm^2^ mg^− 1^), root tissue density (RTD, mg cm^− 3^) were then calculated.

### Root APase activity

Root APase activity of *Alhagi sparsifolia* was determined regarding the methodology developed by Tabatabai and Bremner [38], with some modifications introduced. Prior to this determination, root samples were washed with deionized water and any plant litter attached to them was removed. Briefly, fresh root samples (each 1.0 g) were weighed and placed in a 25-mL centrifuge tube, to which 8-mL of 0.2 M sodium acetate buffer (pH 5.8) was added, and this ground in an ice environment and then filtered and centrifuged for 15 min at 12 000 r min^–1^. Form the supernatant solution, 1-mL was transferred to a new 15-mL centrifuge tube, to which was added 2-mL of 0.05 M *p*-nitrophenyl phosphate (*p*NPP), and this kept in the dark at 37℃ for 30 min. After this incubation, 2-mL of 0.5 M CaCl_2_ and 2-mL of 2 M NaOH were added to stop the reaction. Then, the 15-mL centrifuge tube was centrifuged at 2500 r min^–1^ for 5 min. The ensuing supernatant solution was transferred to a new 15-mL centrifuge tube and centrifuged at 4000 r min^–1^ for 5 min. Finally, a 5-mL aliquot was determined in a spectrophotometer at 410 nm for APase activity, this expressed as µmol *p*NP per gram of root per min (µmol *p*NP g^− 1^ min^− 1^).

### Root P, leaf P and leaf mn concentration

After weighing the root biomass and aboveground (leaf + stem) biomass, the samples of coarse roots, fine roots and leaves were ground, passed through a 0.15-mm sieve, and then digested in a mixture of concentrated H_2_SO_4_, HClO_4_, and HNO_3_ (v:v:v = 1:2:7). To quantify their concentrations of root P, leaf P and leaf Mn in the digest, we used inductively coupled plasma-atomic absorption spectroscopy (ICP-ABS Hitachi Z-5000, Japan).

### Soil hedley P pool

The soil Hedley P pool was subdivided into eight fractions in this study, including resin-P, NaHCO_3_-Pi, NaHCO_3_-Po, NaOH-Pi, NaOH-Po, conc. HCl-Pi, conc. HCl-Po, and residual-P [39]. Briefly, soil samples (1.0 g) were weighed after sieving (< 2 mm) and put in a 50 mL tube. The different extractants were then used to extract each P fraction in soils: deionized water containing 0.5 g ion exchange resin (resin-P); 0.5 mol L^–1^ NaHCO_3_ (NaHCO_3_-Pi and NaHCO_3_-Po); 0.1 mol L^–1^ NaOH (NaOH-Pi and NaOH-Po); hot concentrated HCl (conc. HCl-Pi and HCl-Po); concentrated nitric acid (residual-P). See Gao et al. [1] for a more detailed fractionation process.

### Statistical analysis

Statistical analyses were implemented in the SPSS 21.0. ANOVAs (one-, two-, and three-way) were used to analyze whether the root traits (root morphological traits, root APase activity, root biomass, root P concentration) differed among the root type, root age, and four P supply treatments. One-way ANOVA testing was also used to analyze leaf P concentration and soil Hedley P pool under different P supply treatments. Simple linear regression was used to evaluate the relationship of root APase activity or leaf Mn concentration to SRL, SRSA, and RTD, and root P to leaf P. Relationships among root P concentration, root biomass, leaf Mn concentration, root APase activity, and root morphological traits were assessed by Pearson correlations and their corresponding heatmap. Then, based on the logical relationships between all root traits and leaf Mn concentration, structural equation modeling (SEM) was used to establish a statistical pattern of high matching, to explore the causal relationships between root P concentration and leaf Mn concentration vis-à-vis the other root traits. The SEM fitted in stepwise manner, to only retain pathways with significant coefficients. The SEM analyses were performed using IBM SPSS-Amos 26.0. The fitted models were deemed acceptable according to these criteria: χ^2^ test (*P* > 0.05 indicates the models is accepted), the root-square mean error of approximation (RMSEA < 0.05 indicates a good fit, 0.05 < RMSEA < 0.08 indicates a reasonable fit), goodness-of-fit index (GFI > 0.90), and comparative fit index (CFI > 0.90) [40].

## Results

### Root biomass

**Fig. 1:**
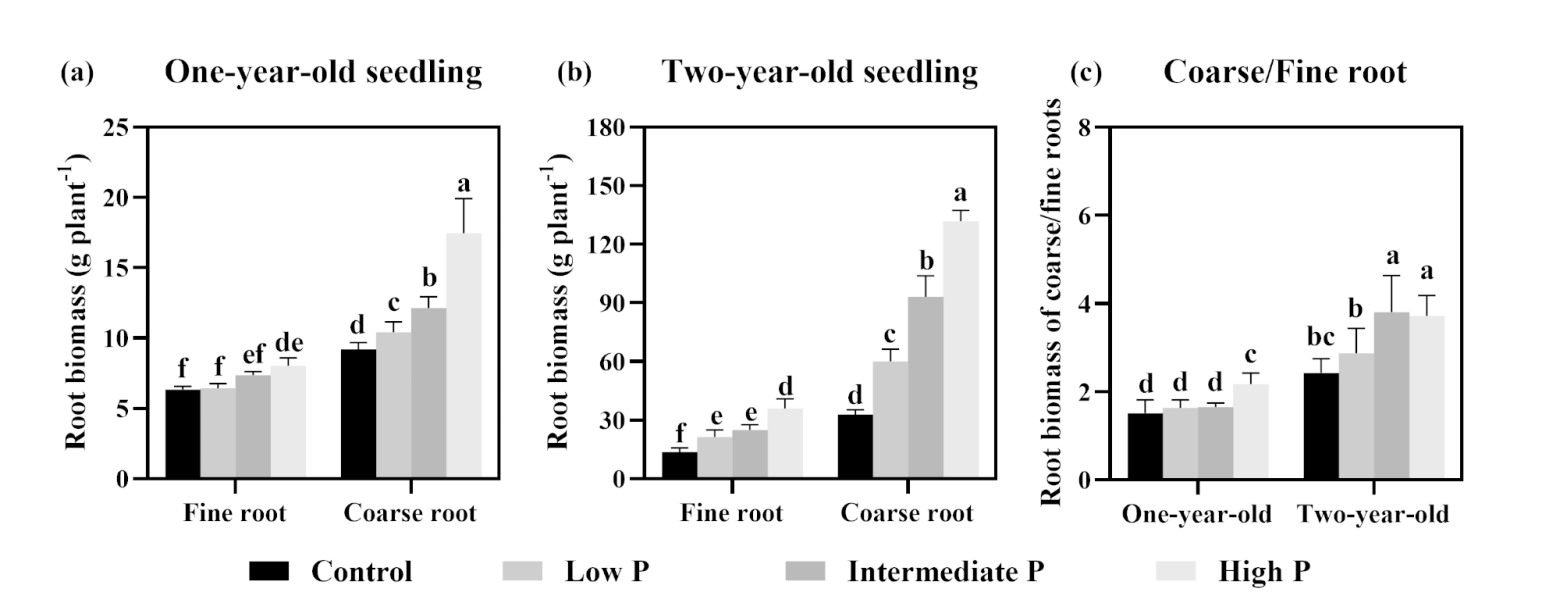
Variation of biomass of the coarse and fine roots at different phosphorus supply levels (Note: Lowercase letters indicate significant differences among soil P levels (*P* < 0.05))

Root biomass (RB) was significantly influenced by root type (*P* < 0.001), root age (*P* < 0.001), P supply treatment (*P* = 0.007) and their interaction (*P* < 0.001, Table 1). The biomass of fine and coarse roots increased significantly with an increasing P supply level (Fig. 1). Compared with the control, the fine root biomass (FRB) and coarse root biomass (CRB) of one-year-old seedlings increased by 26.9% and 89.8% in response to high-P supply (Fig. 1a), while increasing for two-year-old seedlings by 161.2% and 301%, respectively (Fig. 1b). In addition, the ratio of CRB to FRB of one-year-old seedlings was significantly greater in the high-P supply than the other P supply conditions, but for the two-year-old seedlings the ratio was remarkably higher in conditions of intermediate- and high-P supply than low-P supply (Fig. 1c). Furthermore, P supply treatment also significantly increased the aboveground biomass (Fig. S1a). The ratio of aboveground to belowground biomass of one-year-old seedlings significantly increased with increasing P supply levels, whereas it significantly decreased for two-year-old seedling (Fig. S1b).

**Table 1:** ANOVA results for root morphological traits as affected by root type (RT), root age (RA), P supply treatment (P), and their interactions

	RP	RB	APase	RL	SA	RV	SRL	SRSA	RTD
*F*									
RT	71.09	22.23	0.01	271.18	21.43	57.95	165.21	28.01	149.49
RA	24.81	54.69	46.29	8.63	72.37	37.73	17.65	54.52	1.40
P	2.95	4.29	6.02	3.36	3.95	1.73	0.13	0.53	2.50
RT×RA	97.78	38.77	132.45	0.04	111.45	148.01	137.69	35.71	12.07
RT×P	1.13	3.69	0.40	8.30	0.48	1.86	0.49	0.75	0.32
RA×P	0.78	6.13	0.22	0.27	0.81	0.63	0.88	13.02	2.52
RT×RA×P	28.92	93.04	7.40	1.30	5.70	15.13	15.47	0.07	8.02
*P*									
RT	< 0.001	< 0.001	0.935	< 0.001	< 0.001	< 0.001	< 0.001	< 0.001	< 0.001
RA	< 0.001	< 0.001	< 0.001	0.004	< 0.001	< 0.001	< 0.001	< 0.001	0.239
P	0.037	0.007	0.001	0.022	0.011	0.167	0.945	0.664	0.064
RT×RA	< 0.001	< 0.001	< 0.001	0.842	< 0.001	< 0.001	< 0.001	< 0.001	0.001
RT×P	0.34	0.015	0.750	< 0.001	0.695	0.143	0.689	0.526	0.831
RA×P	0.51	0.001	0.884	0.850	0.492	0.597	0.454	< 0.001	0.063
RT×RA×P	< 0.001	< 0.001	< 0.001	0.280	0.001	< 0.001	< 0.001	0.976	< 0.001

Note: DF_RT_= 1, DF_RA_ =1, DF_P_ =3, DF_RT×RA_ = 1, DF_RT×P_ = 3, DF_RA×P_ = 3, DF_RT×RA×P_=3. RP, root phosphorus (g kg^–1^); RB, root biomass (g plant^–1^); APase, root APase (µmol *p*NP g^− 1^ min^− 1^); RL, root length (cm); SRL, specific root length (cm mg^–1^); SA, root surface area (cm^2^); SRSA, specific root surface area (cm^2^ mg^–1^); RV, root volume (cm^3^); RTD, root tissue density (mg cm^–3^)

### Root morphological traits

Root morphological traits varied by root type, root age, P supply treatment, and their interaction (Tables 1 and 2). The interactions with root type, root age, and P significantly influenced the SA, RV, SRL, and RTD (*P* < 0.001, Table 1). Further, as shown in Table 2, P supply significantly increased the RL, SA, and RV of the coarse as well as fine roots. For instance, the RL and SA of the fine roots of one-year-old seedlings were increased respectively by 95.22% and 100.54% in response to the high-P supply relative to the control. The SRL and SRSA of one-year-old seedlings were greatest in intermediate-P supply conditions, yet for two-year-old seedlings they peaked in the control treatment, being generally higher for fine roots than coarse roots. Furthermore, the RTD was maximal under the control treatment, except for the coarse roots of the two-year-old seedlings.

**Table 2:** Variation of root morphological traits at different root ages, root types, and P supply levels

Root age	Root type	P supply levels	RL (cm)	SA (cm^2^)	RV (cm^3^)	SRL (cm mg^− 1^)	SRSA (cm^2^ mg^− 1^)	RTD (mg cm^− 3^)
One-year-old seedling	Fine root	Control	232.51 ± 50.49c	33.45 ± 3.56c	0.73 ± 0.03c	36.90 ± 9.00c	5.30 ± 0.72c	8.70 ± 0.54a
		Low P	328.87 ± 33.01b	48.83 ± 5.00b	0.88 ± 0.04c	51.31 ± 5.11b	7.61 ± 0.58b	7.34 ± 0.55b
		Intermediate P	433.58 ± 36.21a	64.33 ± 6.74a	1.22 ± 0.09b	58.99 ± 6.17a	8.73 ± 0.86a	6.04 ± 0.50c
		High P	453.90 ± 23.61a	67.08 ± 3.78a	1.41 ± 0.12a	56.74 ± 5.67a	8.36 ± 0.47a	5.74 ± 0.56d
	Coarse root	Control	34.37 ± 3.82c	21.10 ± 3.02d	1.39 ± 0.18d	3.74 ± 0.44b	2.31 ± 0.41d	6.73 ± 1.11a
		Low P	51.56 ± 5.57b	48.66 ± 2.82c	3.76 ± 0.34c	4.98 ± 0.71a	4.71 ± 0.55b	2.78 ± 0.29b
		Intermediate P	55.57 ± 3.88b	61.82 ± 6.68b	6.23 ± 0.48b	4.60 ± 0.52a	5.09 ± 0.37a	1.96 ± 0.25c
		High P	63.66 ± 2.81a	69.11 ± 2.45a	6.79 ± 0.25a	3.71 ± 0.56b	4.04 ± 0.69c	2.57 ± 0.34b
Two-year-old seedling	Fine root	Control	352.50 ± 36.85d	59.74 ± 3.09c	1.46 ± 0.50c	25.96 ± 4.34a	4.41 ± 0.64a	10.42 ± 3.85a
		Low P	410.47 ± 63.72c	66.90 ± 9.45b	2.91 ± 0.40b	19.78 ± 5.41b	3.21 ± 0.78b	7.38 ± 0.85d
		Intermediate P	486.47 ± 58.54b	73.79 ± 9.61b	3.04 ± 0.49b	19.55 ± 1.90b	2.99 ± 0.50c	8.36 ± 1.63c
		High P	601.33 ± 43.25a	119.05 ± 12.90a	3.80 ± 0.47a	16.97 ± 2.74c	3.38 ± 0.73b	9.53 ± 1.39b
	Coarse root	Control	117.94 ± 21.32c	139.48 ± 21.26d	16.65 ± 0.76c	3.63 ± 0.82a	4.28 ± 0.82a	1.98 ± 0.15c
		Low P	141.18 ± 22.32b	184.86 ± 22.55c	21.34 ± 1.65b	2.38 ± 0.51b	3.10 ± 0.46b	2.83 ± 0.34b
		Intermediate P	149.21 ± 27.89b	219.81 ± 11.95b	32.01 ± 4.88a	1.61 ± 0.29c	2.38 ± 0.19c	2.97 ± 0.64b
		High P	223.47 ± 27.47a	235.87 ± 25.39a	31.62 ± 2.77a	1.70 ± 0.28c	1.79 ± 0.24d	4.20 ± 0.41a

Note: Lowercase letters indicate differences among different soil P levels (*P* < 0.05). RL, root length; SA, root surface area; RV, root volume; SRL, specific root length; SRSA, specific root surface area; RTD, Root tissue density

### Root APase activity and leaf Mn concentration

**Fig. 2:**
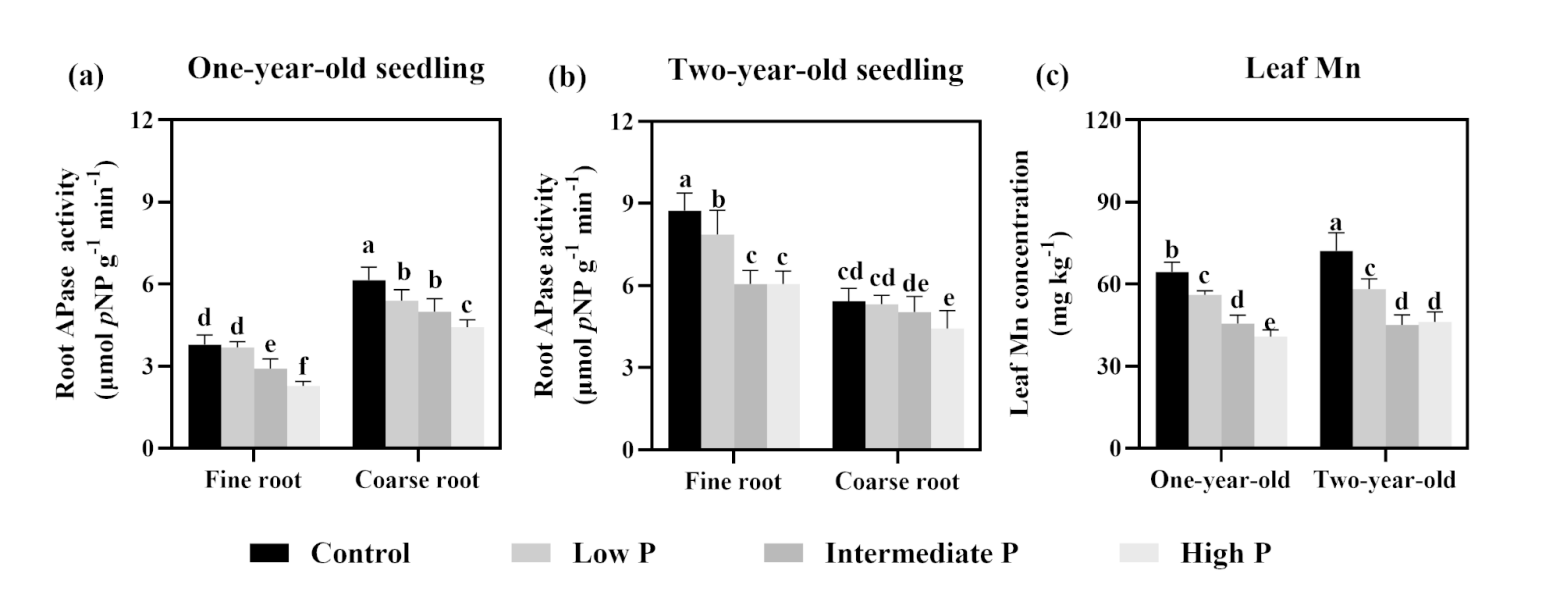
Variation of root acid phosphatase activity and leaf Mn concentration at different phosphorus supply levels (Note: Lowercase letters indicate significant differences among soil P levels (*P* < 0.05))

Root APase activity was significantly influenced by root age (*P* < 0.001), P supply treatment (*P* = 0.001) and the interaction between root type, P supply treatment and root age (*P* < 0.001). Increased the P supply significantly reduced the APase activity of both fine and coarse roots and also decreased the leaf Mn concentration (Fig. 2). Compared with the high-P supply treatment, the APase activity of fine and coarse roots of one-year-old seedlings was significantly increased by 66.39% and 38.78% under the control treatment, and by 44.11% and 22.79% for two-year-old seedlings, respectively. (Fig. 2a, b). Moreover, in response to either the control or low-P supply treatment, the leaf Mn concentration increased. For instance, the leaf Mn concentration of one-year-old and two-year-old seedlings increased considerably, by 58.40% and 56.43% respectively, in the control relative to high-P supply condition (Fig. 2c).

### Root and leaf P concentration

**Fig. 3:**
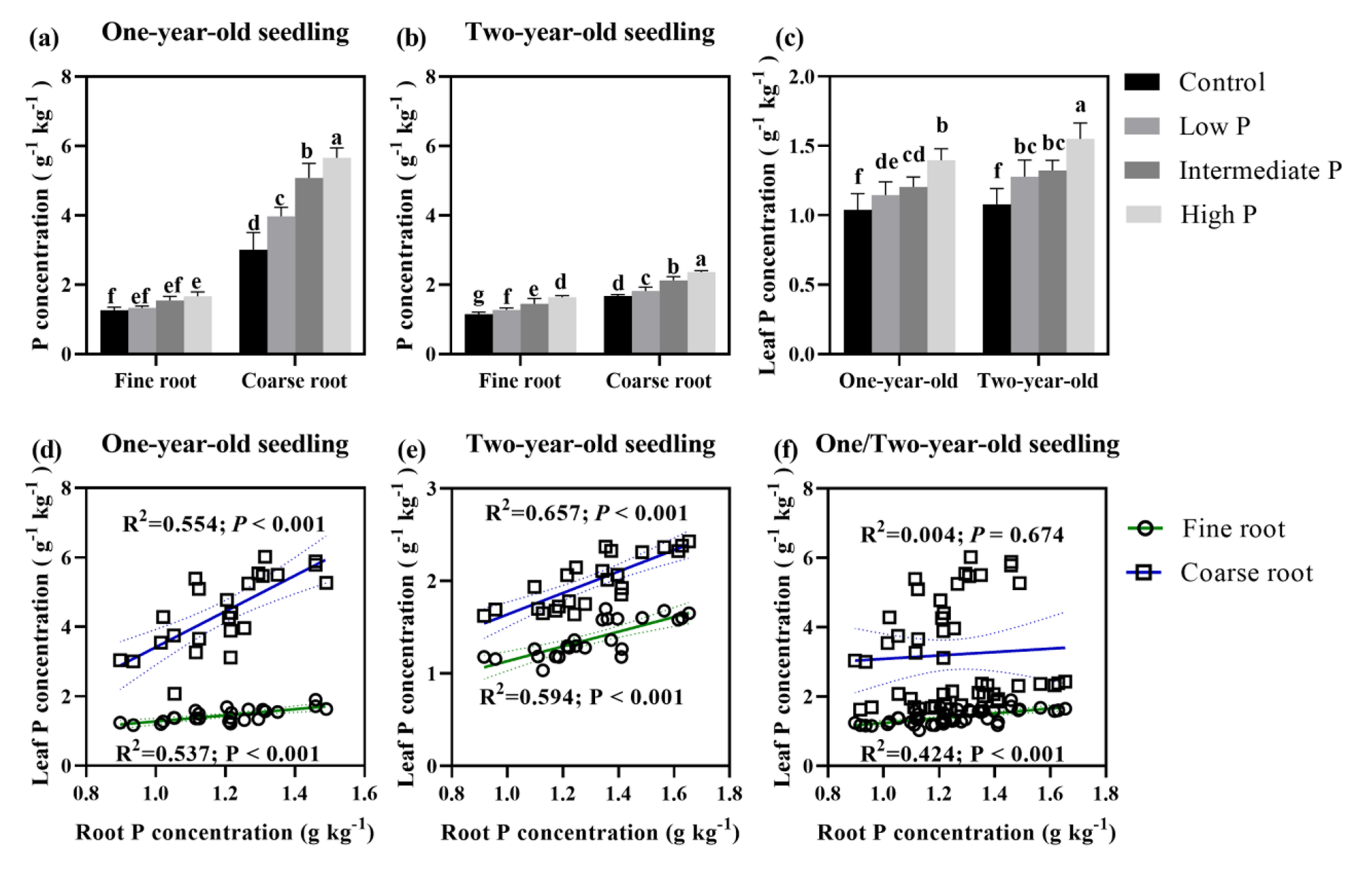
Variation of root and leaf phosphorus concentration at different phosphorus supply levels (Note: Lowercase letters indicate significant differences among soil P levels (*P* < 0.05))

Supplying more P significantly increased the P concentration of leaf, coarse and fine roots (Fig. 3). For instance, the P concentration in the fine and coarse roots of one-year-old seedlings was respectively increased by 31.90% and 88.11% in response to the high-P supply relative to the control (Fig. 3a), for two-year-old seedlings, corresponding increases were 41.91% and 40.88%, respectively (Fig. 3b), for leaf P concentration of one- and two-year-old seedlings, corresponding increases were 34.47% and 43.72%, respectively (Fig. 3c). Leaf P concentration of one- and two-year-old seedlings were positively related to their fine and coarse P concentration (Fig. 3d, e). Generally, leaf P concentration was significantly positively correlated with fine root P concentration (Fig. 3f). Moreover, the RP concentration differed significantly between root type (*P* < 0.001), root age (*P* < 0.001), and was affected by their interaction (*P* < 0.001). Furthermore, the mean P concentration of fine and coarse roots across all P supply levels was 1.45 and 4.43 g kg^− 1^ for one-year-old seedlings and 1.38 and 1.99 g kg^− 1^ for two-year-old seedlings, respectively. This indicated that a dilution effect occurred in the coarse root P concentration as the seedlings grew.

### Soil P fractions

**Fig. 4:**
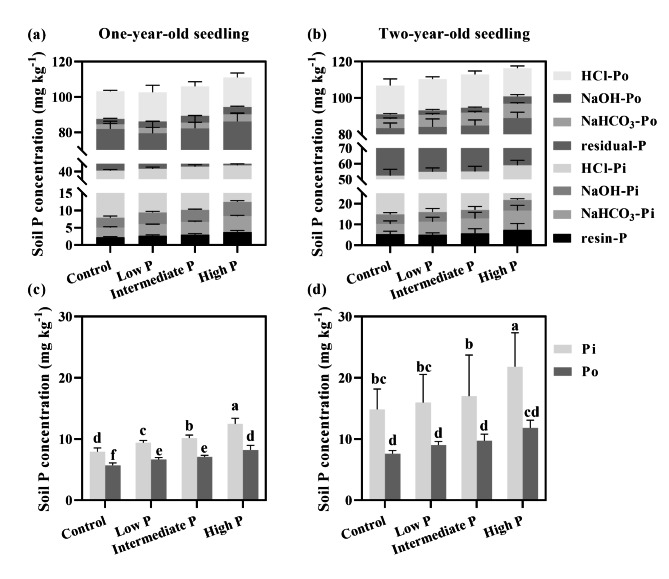
Variation of soil Hedley phosphorus fractions at different root ages and phosphorus supply levels (Note: Lowercase letters indicate significant differences among soil P levels (*P* < 0.05). Inorganic phosphorus (Pi) fractions include resin-P, NaHCO_3_-Pi, NaOH-Pi, and conc. HCl-Pi. Organic phosphorus (Po) includes NaHCO_3_-Po, NaOH-Po, and conc. HCl-Po)

The concentration of soil Pi (resin-P, NaHCO_3_-Pi, and NaOH-Pi) and Po (NaHCO_3_-Po and NaOH-Po) increased with an increasing P supply level, except conc. HCl-P and residual-P (Table S3; Fig. 4). The concentrations of Pi and Po in the rhizosphere soil of two-year-old seedlings were higher than that of one-year-old seedlings. Especially, the variation of soil Po (NaHCO_3_-Po and NaOH-Po) of two-year-old seedlings was greater than that of Pi (NaHCO_3_-Pi). Compared to the control (no P supply), soil Pi and Po of one-year-old seedlings increased by 63.0% and 44.0% in response to high-P supply, while increasing for two-year-old seedlings by 57.6% and 55.4%, respectively (Fig. 4).

### Correlations between root morphological traits and root APase activity and leaf mn concentration

**Fig. 5:**
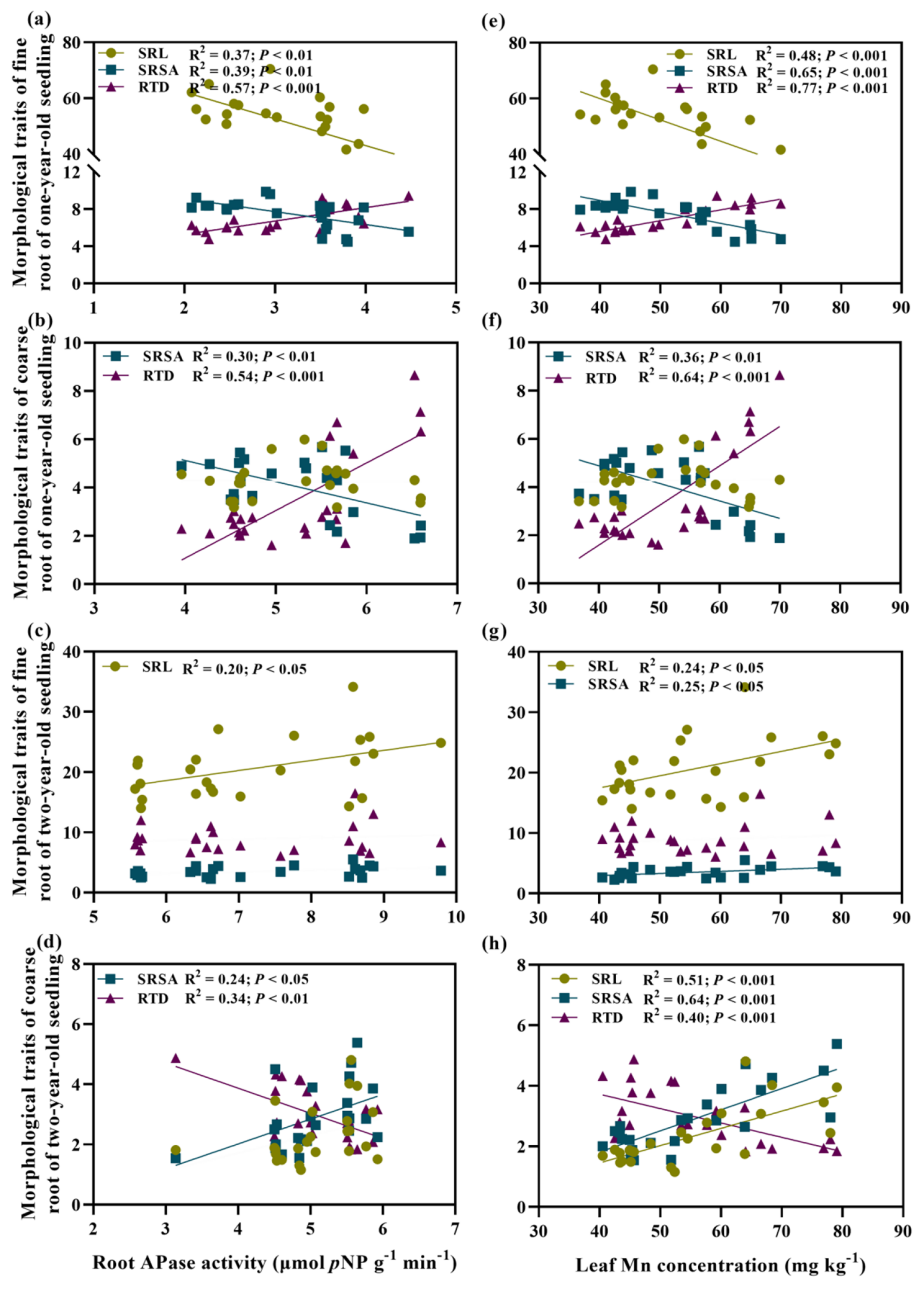
Correlations between root morphology traits, acid phosphatase activity and leaf Mn concentration (Note: Solid lines indicate only those relationships that are significant (i.e., *P* < 0.05). SRL, specific root length; SRSA, specific root surface area; RTD, root tissue density)

Root morphological traits (SRL, SRSA, and RTD) were tested for their correlation with the root APase activity and leaf Mn concentration (Fig. 5). Leaf Mn concentration of one-year-old seedlings and the APase activity of their coarse and fine roots increased with increasing RTD (*P* < 0.001) but decreased with an increasing SRSA (*P* < 0.01) (Fig. 5a, b, e, f). In addition, for one-year-old seedlings, their leaf Mn concentration and APase activity of their fine roots increased with decreasing SRL (*P* < 0.01), and vice versa for the two-year-old seedlings (Fig. 5c, g). For two-year-old seedlings, the RTD of coarse roots was negatively related, but the SRSA was positively related, to their root APase activity and leaf Mn concentration.

### Variation in root traits and leaf mn concentration were coordinated with root P concentration

**Fig. 6:**
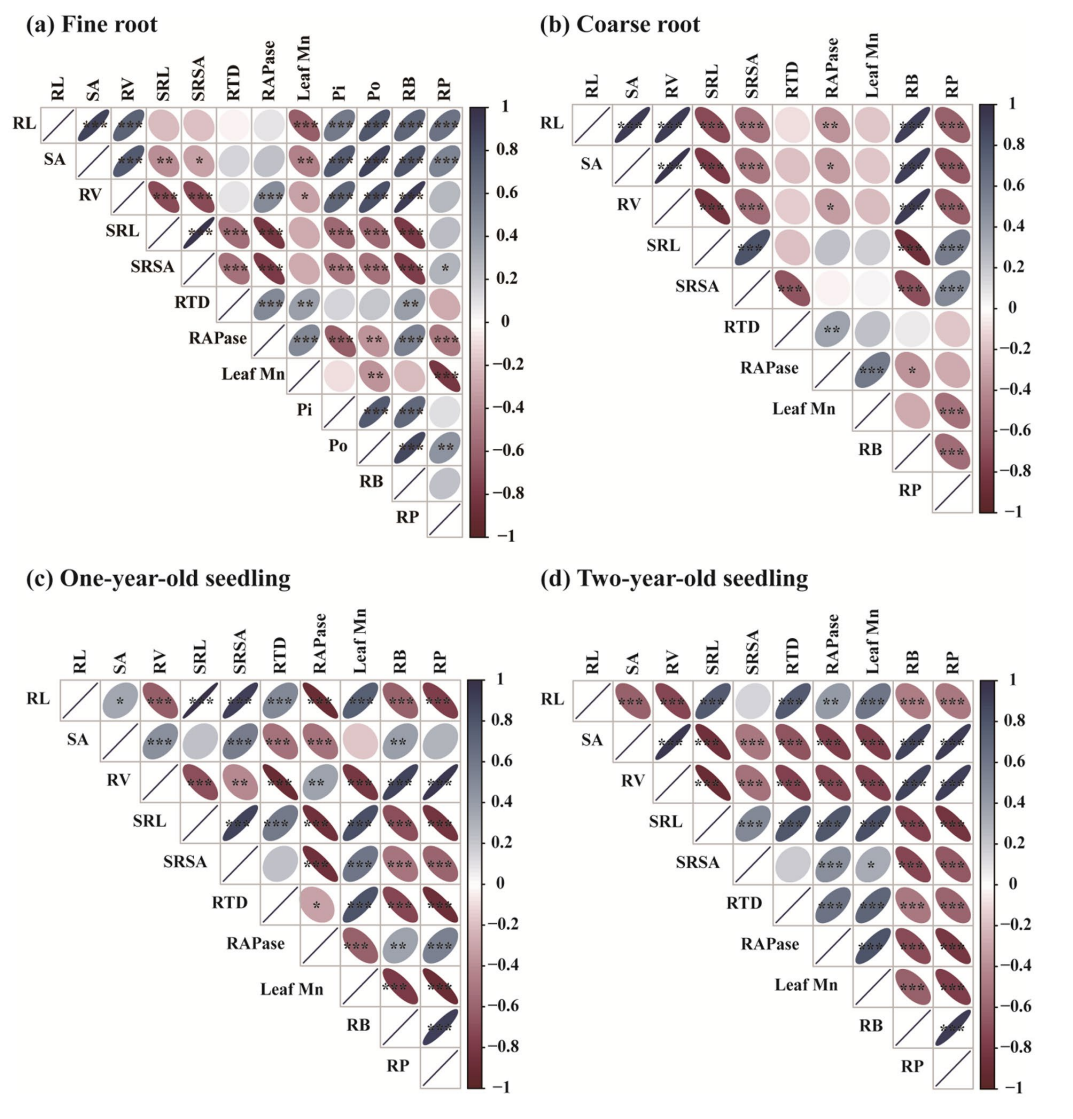
Correlations between root morphology traits, acid phosphatase activity, leaf Mn concentration, root biomass, and root P concentration (Note: Data from one- and two-year-old seedlings were used for both fine and coarse root analyses. Data for both one- and two-year-old seedlings were analyzed using both coarse and fine roots. A blue ellipse indicates a positive correlation, and a red ellipse indicates a negative correlation. The shape and color depth of the ellipse represent the absolute value of the correlation, and the flatter the ellipse and the darker the color, the greater the absolute value of the correlation. Asterisks denote significance levels (* *P* < 0.05, ** *P* < 0.01, *** *P* < 0.001). RL, root length (cm); SRL, specific root length (cm mg^–1^); SA, root surface area (cm^2^); SRSA, specific root surface area (cm^2^ mg^–1^); RV, root volume (cm^3^); RTD, root tissue density (mg cm^–3^); RB, root biomass (g plant^–1^); RAPase, root APase (µmol *p*NP g^− 1^ min^− 1^); RP, root phosphorus (g kg^–1^); Pi, soil inorganic P; and Po, soil organic P)

**Fig. 7:**
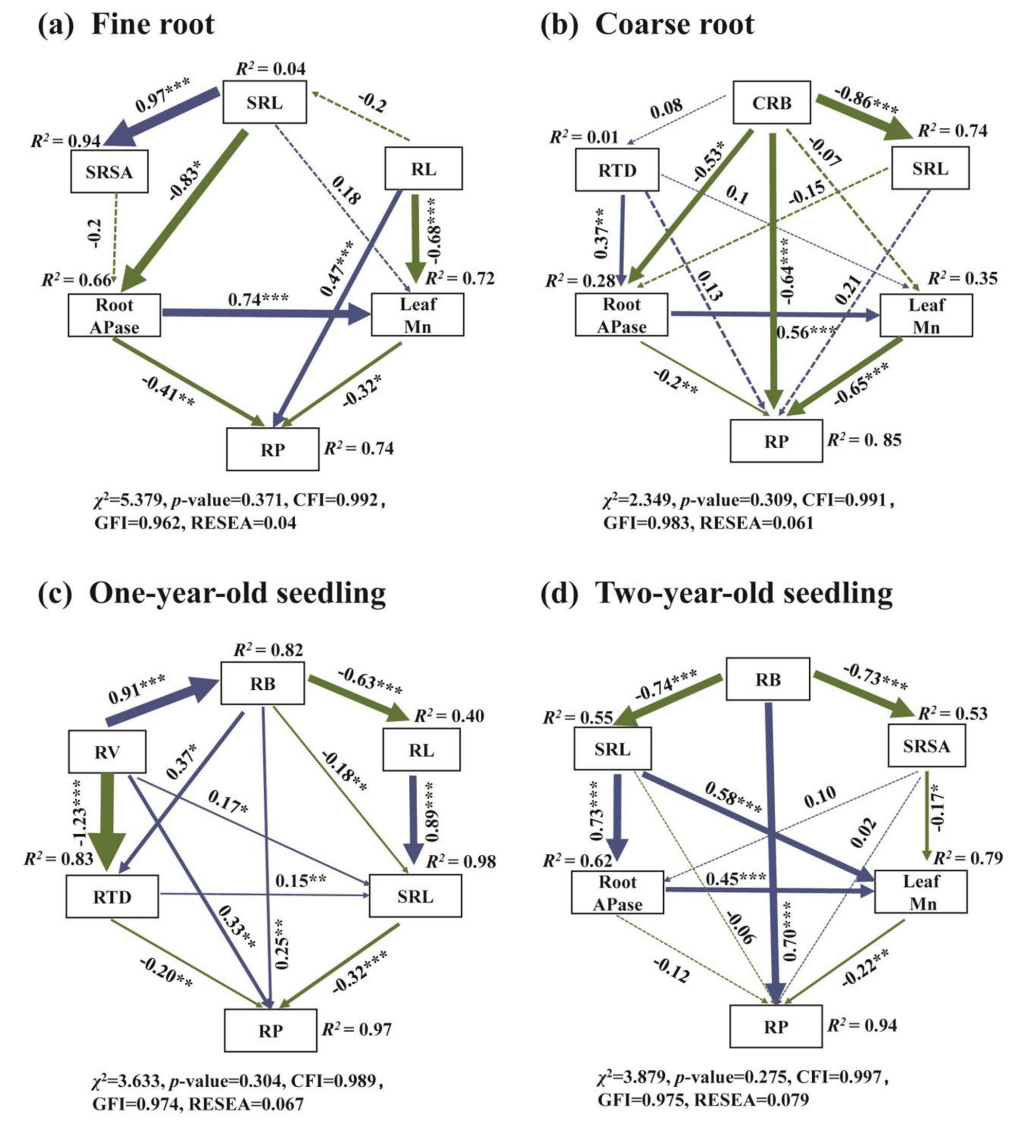
Structural equation models of root traits and root phosphorus concentration (Note: Blue lines indicate positive relationships, and green lines indicate negative relationships. Solid lines represent significant relationships and dashed lines represent non-significant relationships. Asterisks represent the level of significance: * not present, *P* > 0.05; otherwise * *P* < 0.05, ** *P* < 0.01, *** *P* < 0.001. Standardized regression coefficients for each path are given, and results for model fit tests are also reported underneath each figure. RP, root phosphorus (g kg^–1^); RL, root length (cm); SRL, specific root length (cm mg^–1^); SRSA, specific root surface area (cm^2^ mg^–1^); RTD, root tissue density (mg cm^–3^); CRB, coarse root biomass (g plant^–1^); RB, root biomass (g plant^–1^); RV, root volume (cm^3^); and RAPase, root APase (µmol *p*NP g^− 1^ min^− 1^))

Fine roots’ RP concentration was closely and positively related to RL, SA and soil Po, but negatively related to both root APase activity and leaf Mn concentration (Fig. 6a). Coarse roots’ RP concentration was significantly positively related to SRL and SRSA, yet negatively related to RL, SA, RV, RB, and leaf Mn concentration (Fig. 6b). In addition, for one-year-old seedlings, their RP concentration was strongly associated with RB, RV, and root APase activity, but negatively associated with RL, SRL, SRSA, RTD, and leaf Mn concentration (Fig. 6c). For two-year-old seedlings, their RP concentration was positively associated with the SA, RV and RB, but negatively associated with RL, SRL, SRSA, RTD, root APase activity, and leaf Mn concentration (Fig. 6d). The SEM indicated that RL (0.47), root APase activity (–0.41), and leaf Mn concentration (–0.32) were the three direct and significant factors that determined the fine roots’ RP concentration (Fig. 7a). Leaf Mn concentration (–0.65), CRB (–0.64), and root APase activity (–0.2) were the three direct and strongest determinants of coarse roots’ RP concentration (Fig. 7b). However, for one-year-old seedlings, RV (0.33), RB (0.25), SRL (–0.32), and RTD (–0.20) were the four direct and significant factors that determined the RP concentration (Fig. 7c). Both RB (0.70) and leaf Mn concentration (–0.22) were the direct and strongest determinants of the RP concentration in two-year-old seedlings (Fig. 7d).

## Discussion

### Variation in root morphological traits, root phosphatase activity, and leaf Mn concentration under differing soil P availability

In this study, as the P supply increased, we found that both SRL and SRSA of one-year-old seedlings increased, whereas those of two-year-old seedlings decreased (Table 1). Moreover, RL, SA, and RV increased as well, but RTD, root APase activity, and leaf Mn concentration all decreased with more P supplied vis-à-vis the control. These results are consistent with those reported for 16-month-old *Syzygium castaneum* [24] and 6-week-old white lupin seedlings [41]. Furthermore, there were differences in the relationships between root morphological traits and root APase activity and leaf Mn concentration at different growth stages, and root APase activity was significantly positively correlated with the leaf Mn concentration (Fig. S2; Figs. 5 and 6). Given these results, our first hypothesis was opposed.

That the soil available P concentration is negatively related to root APase activity and carboxylates concentration is generally reported [13]. This study also suggested that root APase activity and leaf Mn concentration were significantly positively correlated, but root APase activity and leaf Mn concentration were significantly negatively correlated with soil Po and Pi (Fig. 6). It indicated that P-activation strategies of Po mobilization and Pi dissolution co-exist in the root system of *Alhagi sparsifolia*. This is mainly because several plant species may develop more than one P-activation strategy, such as increasing root APase activity and releasing more rhizosphere carboxylates to activate the P adsorbed on soil particles, including Po, in a P-deficient environment [30, 31]. Accordingly, the potential mechanism responsible for the negative correlation of soil available P concentration to root APase activity or the leaf Mn concentration of one- and two-year-old seedlings found in this study is that *Alhagi sparifolia*, acting as a typical legume plant, improved its root APase activity and rhizosphere carboxylates’ concentration to acquire more P when the soil available P supply is locally reduced [1]. Moreover, root APase activity of the control *Alhagi sparifolia* seedlings exceeded that of 6-week-old white lupin and 16-month-old *Syzygium castaneum* seddlings [24, 41], which also strongly confirms the P-impoverished state of in soils in our study area [1, 7]. Furthermore, the results of SEM showed that carboxylates played a more important role in root P-acquisition than root APase (Fig. 7). It suggested that the exudation of phosphatase by plants is indeed a more resource-costly strategy than carboxylates exudation [13].

Root morphological traits generally determine the adaptability and acquisition ability of plant roots to limiting nutrients [42]. For instance, a higher RL and SA and a lower root diameter in a P-deficient environment can contribute P assimilation by augmenting the interaction surface between root and soil solid phase [43]. However, we found that both RL and SA of coarse and fine roots of *Alhagi sparsifolia* seedlings were lower in the control than in high-P supply treatment (Table 2). Interestingly, for 1-year-old seedlings their SRL and SRSA were both higher under the three P supply treatments than the control condition, but two-year-old seedlings the opposite response was found, being greater for fine roots than coarse roots (Table 2). This suggests the higher SRL and SRSA of fine roots enabled them to explore a larger soil volume and increase their interaction surface area with soil, so that ultimately the roots acquired more P [13, 27]. In addition, for plants whose roots grow in a chronic P-deficient environment these tend to be of the resource acquisition type (e.g., having higher SRL and SRSA), this as demonstrated in earlier studies [43, 44]. Alternatively, due to the low lignification degree of roots and a larger proportion of roots able to absorb nutrients, the root traits of one-year-old seedlings could have responded rapidly to soil P availability [17, 45]. For instance, this study found that soil Pi and Po concentrations were more closely related to the fine root traits of one-year-old seedlings than two-year-old seedlings (Fig. S2a, c). Therefore, the SRL and SRSA of two-year-old seedlings were substantially lower under P supply treatments than the control and clearly positively correlated with root APase activity and leaf Mn concentration, but one-year-old seedlings had a completely opposite pattern.

In addition, having a high RTD in condition of low P is generally considered a key adaptation strategy to soil P deficiency [42]. For example, the fine roots’ RTD was generally highest in the control treatment, not the P supply treatments (Table 2). This result implied that a higher RTD slows down the growth rate of roots and increases the tissue extension and defense ability (e.g. pathogen resistance) of plants growing under P-deficient conditions [46]. Consequently, a high RTD in a low P environment may favor the production of fine roots, thereby further increasing their ability to acquire water and limiting nutrients [46, 47]. Additionally, higher RTD often entails a larger root biomass per unit root volume (C-investment) [48]. Other research has found that thinner roots have higher C maintenance costs and display higher respiration and turnover rates than do coarse roots [17]. On the one hand, the high C-investment produces roots whose SRL and SRSA are enhanced, accompanied by a high risk of C-loss, which can be compensated by high P-uptake facilitated by an increased content of extracellular phosphatase and carboxylates [24]. On the other hand, the RTD of two-year-old seedlings was negatively related to their root APase activity and leaf Mn concentration, a result similar to that of Lugli et al. [13]. Therefore, the negative correlations between RTD and root APase activity and leaf Mn concentration can be interpreted as a strategy to maximize the investment of belowground biomass per unit RV, thus saving more energy for the release of rhizosphere carboxylates and APase, to improve the P acquisition by roots [49].

### Trade-offs between root traits and root P-acquisition strategies under a P-deficient environment

The correlations between the RP concentration and root traits at different growth stages could be a part of the ‘slow–fast’ trait syndrome proposed by Reich [50], where plant functional traits are coordinated to acquire and conserve limited nutrients in a nutrient-deficient environment. Our results showed that the RP concentration of one- and two-year-old seedlings was significantly negatively related to their RL, SRL, SRSA, RTD, root APase activity and leaf Mn concentration—except for the root APase activity of one-year-old seedling—but significantly positively related to SA, RV, and RB (Fig. 6). A similar pattern was observed in other plant species (e.g., *Folium subterraneum*, *Lupinus albus* or *Hakea prostrata*) and ecosystems (e.g., forest, grassland or farmland) analyzed for root traits and RP concentration [30, 51]. Our results implied that root traits of *Alhagi sparsifolia* seedlings at different growth stages are coordinated to acquire P nutrients under P-deficient soils, in support of our second hypothesis.

Careful explication of the coordination between root traits and RP concentration at different growth stages in response to the P supply treatments is needed, because we explored root traits and RP concentrations of the coarse and fine roots, respectively. As the most active interface of material and energy exchange with the external environment, fine roots are often considered starkly different from the coarse roots in terms of nutrient and water acquisition strategies [48, 52]. This is mainly due to the divergence of root traits between coarse and fine roots [53]. The construction and maintenance of fine roots are considered to be high C-cost functions [17, 21], suggesting there should be a stronger relationship between root nutrient concentration (e.g., RP) and root traits (e.g., SA, root biomass, and APase activity) in fine roots than coarse roots [54]. On the contrary, CRB was the direct and strongest determinant for the RP concentration of coarse roots in this study, but there was no significant relationship between FRB and fine root P concentration (Figs. 6 and 7). A possible explanation is that a reduction in FRB is expected when more P is supplied as a sign of P limitation becoming alleviated [21]. Contrary to that, this study demonstrated that FRB actually increased under a greater P supply (Fig. 1), which conflicts with the findings of Yavitt et al. [55] in a lowland tropical moist forest, but is consistent with research in a tropical dry secondary forest and P-deficient and P-fertility soils in Amazonia [56]. Coarse roots feature a small surface area of absorbable nutrients per unit biomass when compared with fine roots, thus the CRB and P concentration of coarse roots was significantly negatively correlated. However, both one- and two-year-old seedlings had an RP concentration that was positively associated with their RB, which may imply that the RB of the seedling stage improves RP acquisition via direct or indirect ways [54]. Moreover, the correlation between RB and RP of two-year-old seedlings (0.70) was much stronger than that of one-year-old seedlings (0.25) (Fig. 7). This disparity may be attributed to the increase in the proportion of RB to the whole plant biomass of two-year-old seedlings (Fig. S1). The consistency of our findings with the above-cited studies suggests that a common response to higher P concentrations and to experimental manipulation. However, our results also suggest that *Alhagi sparifolia* root biomass changes rapidly with a greater P supply, indicating an enhanced effect of root nutrients acquired under P-fertile conditions.

Another critical point is that although direct examination of whole root traits of perennial desert species are more convincing than a reliance upon partial root samples, there are some difficulties in obtaining entire roots in the natural ecosystem, especially for deep-rooted desert species [12, 17]. According to a recent study, young and adult individuals of plant species present intraspecific differences in the correlation between root traits and nutrient-acquirement strategy [51]. The present study also confirms that the response of RP concentration to root traits is not the same between one- and two-year-old seedlings (Figs. 6 and 7). A potential mechanism may be that as roots grow, their degree of lignification increases, which decreases the ability of roots to uptake nutrients and water [17]. Alternatively, reducing the proportion of roots capable of obtaining nutrients will result in new trade-offs between root traits and P-acquisition strategies [22, 45]. Generally, *Alhagi sparsifolia* seedlings have different root P-acquisition strategies at different growth stages and are mainly affected by different root traits. Among them, however, RB and carboxylates are important root traits that should be emphasized in the engineering of *Alhagi sparsifolia* as a windbreak and sand-fixing in this plant’s cultivation in deserts. In addition, the present study also suggested that the variations of P in *Alhagi sparsifolia* leaves and roots in different P availability soils had the same trend, especially between leaves and fine roots (Fig. 3). Therefore, for desert deep-rooted plants with large roots in the natural ecosystem that are some difficulties in obtaining fine roots, the leaf P may be a good substitute for exploring the P-acquisition by fine roots.

## Conclusions

This study shows that, in the P-deficient desert ecosystem, physiological and morphological traits of fine and coarse roots of one- and two-year-old seedlings of a desert species can change with increasing soil P supply treatments, whose morphological traits are closely correlated to leaf Mn concentration and root APase activity. But there were discrepancies in the trade-offs between root traits and P-acquisition strategies of different growth stages and root types. In the control or low P supply conditions, high root APase activity and leaf Mn concentration are conducive to acquiring more P from P-deficient soils. It implied that *Alhagi sparsifolia* had both phosphatase and carboxylates P-activation strategy. The specific root length and specific surface area for two-year-old seedlings were also higher in the control than P supply treatment, but the specific root length and specific surface area for one-year-old seedlings increased with greater soil P availability. The variation in root traits of different growth stages and root types are coordinated with the variation of root P concentrations, which indicates that root linkages exist between root physiological and morphological traits and P-acquisition strategies. Furthermore, the present study suggested that P-activation strategies of organic P mobilization and inorganic dissolution co-exist in the root system of *Alhagi sparsifolia*. Among them, carboxylate released by roots was the main driving force for activating P. The adaptive changes in the P-acquisition strategies may be conducive to maintaining the desert ecosystem productivity at the junction of the southern edge of the Taklimakan desert.

## Electronic supplementary material

Below is the link to the electronic supplementary material.

Supplementary Material 1

## Data Availability

The datasets supporting the findings of this article are included within the article and its additional files. The data are available from the corresponding author on reasonable request.

## Abbreviations

RL: Root length
SRL: Specific root length
SA: Root surface area
SRSA: Specific root surface area
RV: Root volume
RTD: Root tissue density
RB: Root biomass
APase: Root acid phosphatase activity
RP: Root phosphorus
