# Supplementary material for: Root morphological and physiological traits are committed to the phosphorus acquisition of the desert plants in phosphorus-deficient soils
Source: BMC Plant Biol. 2023 Apr 10;23:188. doi: 10.1186/s12870-023-04178-y (PMC10084647; doi:10.1186/s12870-023-04178-y)
Supplement: Supplementary file 1 — Supplementary Material 1 [file 12870_2023_4178_MOESM1_ESM.doc]

**Table S1 The physicochemical properties of the tested soil**

| Soils properties | Aeolian sandy |
| --- | --- |
| pH^a^ | 8.63±0.03 |
| EC^b^ (μs cm^–1^) | 292.67±3.51 |
| SOM^c^ (mg kg^–1^) | 4.66±0.09 |
| Olsen-P^d^ (mg kg^–1^) | 3.46±0.07 |
| NH_4_^+^-N^e^ (mg kg^–1^) | 1.39±0.09 |
| NO_3_^–^-N^f^ (mg kg^–1^) | 13.07±0.26 |
| Available-K^g^ (mg kg^–1^) | 136.33±1.15 |
| Total-P^h^ (g kg^–1^) | 0.63±0.02 |
| Total-N^i^ (g kg^–1^) | 0.22±0.01 |
| Total-K^j^ (g kg^–1^) | 17.41±0.23 |
| Bulk density^k^ (g cm^–3^) | 1.34±0.06 |

Note: Data are the mean ± standard deviation (SD)

^a^ pH was measured using a 1:5 ratio of soil to Nanopure water.
^b^ EC was measured using a 1:5 ratio of soil to Nanopure water.
^c^ SOM was measured by the wet-oxidation technique.

^d^ Olsen-P was measured by the Olsen method.
^e^ NH_4_^+^-N and ^f^ NO_3_^–^-N were measured using a 1M KCl as the extractant, then analyzed by an AA3 continuous flow analytical system (CFA) of soil to Nanopure water.
^g^ Available-K and ^j^ Total-K was measured by extraction with ammonium acetate.

^h^ Total-P were measured by the perchloric acid digestion method.

^i^ Total-N was extracted with 5 mL concentrated H_2_SO_4_ at 360 C and then analyzed using the semi-automatic Kjeldahl method.

^k^ Bulk density was measured by the cutting ring method.

Table S2 Soil phosphorus fractions of the tested soil

|  | P fractions (mg kg^-1^) |
| --- | --- |
| resin-P | 2.31±0.11 |
| NaHCO_3_-Pi | 2.50±0.15 |
| NaHCO_3_-Po | 2.83±0.44 |
| NaOH-Pi | 4.04±0.46 |
| NaOH-Po | 4.42±1.21 |
| conc. HCl-Pi | 31.75±3.66 |
| conc. HCl-Po | 15.58±2.31 |
| residual-P | 37.66±3.88 |

Note: Data are the mean ± standard deviation (SD).

**Table S3 Variation of soil Hedley phosphorus fractions at** **different root ages and phosphorus supply levels.**

| Root age | P supply levels | resin-P | NaHCO_3_-Pi | NaOH-Pi | conc. HCl-Pi | residual-P | NaHCO_3_-Po | NaOH-Po | conc. HCl-Po |
| --- | --- | --- | --- | --- | --- | --- | --- | --- | --- |
| One-year-old  seedling | Control | 2.25±0.21c | 2.79±0.26d | 2.89±0.47c | 33.42±1.24b | 40.55±4.44a | 2.63±0.47c | 3.09±0.31c | 15.62±0.58a |
|  | Low P | 2.70±0.24b | 3.32±0.05c | 3.39±0.31b | 34.12±2.25ab | 35.94±6.67a | 2.99±0.33bc | 3.68±0.16b | 16.52±4.02a |
|  | Intermediate P | 3.02±0.32b | 3.61±0.30b | 3.54±0.22b | 36.22±0.92a | 35.84±7.23a | 3.22±0.22b | 3.87±0.3ab | 16.72±2.54a |
|  | High P | 3.77±0.44a | 4.55±0.27a | 4.23±0.44a | 35.43±2.08ab | 38.21±4.77a | 4.10±0.46a | 4.15±0.42a | 16.74±2.53a |
| Two-year-old  seddling | Control | 5.36±1.42a | 4.87±1.53b | 4.64±0.81a | 37.67±3.97a | 30.94±2.76a | 4.94±0.63c | 2.67±0.40b | 15.73±3.67a |
|  | Low P | 5.10±0.86a | 6.17±2.24b | 4.69±1.75a | 38.95±2.49a | 29.28±4.32a | 6.50±0.87b | 2.53±0.49b | 17.17±1.27a |
|  | Intermediate P | 5.69±2.29a | 7.14±2.98ab | 4.18±1.63a | 38.12±3.39a | 29.82±2.96a | 7.00±0.79ab | 2.75±0.34b | 18.21±1.96a |
|  | High P | 7.43±3.07a | 9.29±2.52a | 5.10±0.56a | 37.22±3.31a | 30.02±3.13a | 7.75±0.65a | 4.06±0.94a | 15.48±1.23a |

Note: Lowercase letters indicate differences among different soil P supply levels (P < 0.05).

**Figure S1 Aboveground biomass and the ratio of aboveground biomass to root biomass.**

**
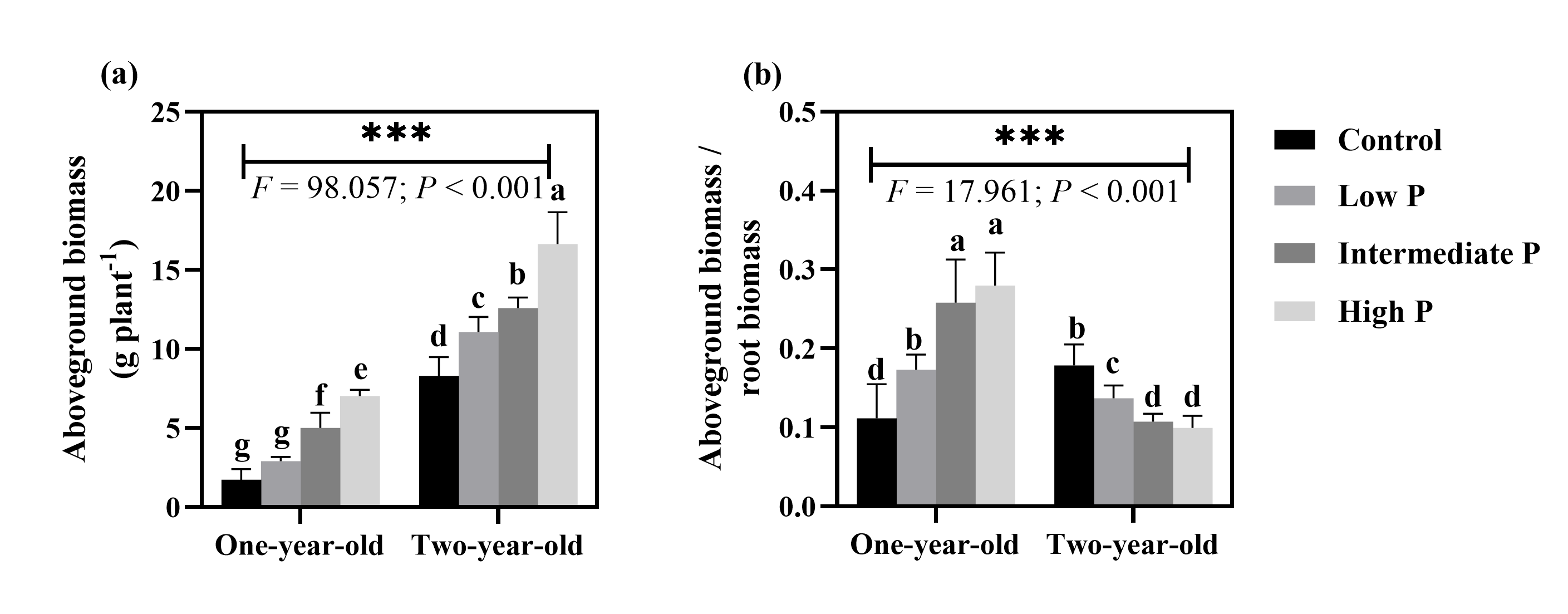
**

Note: Lowercase letters indicate significant differences among soil P levels (*P* < 0.05). Asterisks indicate significantly differences between one-year-old and two-year-old seedling. *** *P* < 0.001.

**Figure S2 Correlations between root morphology traits, acid phosphatase activity, leaf Mn concentration, root biomass, root P concentration and soil Pi and Po of fine and coarse root of one- and two-old-year-seedling.**


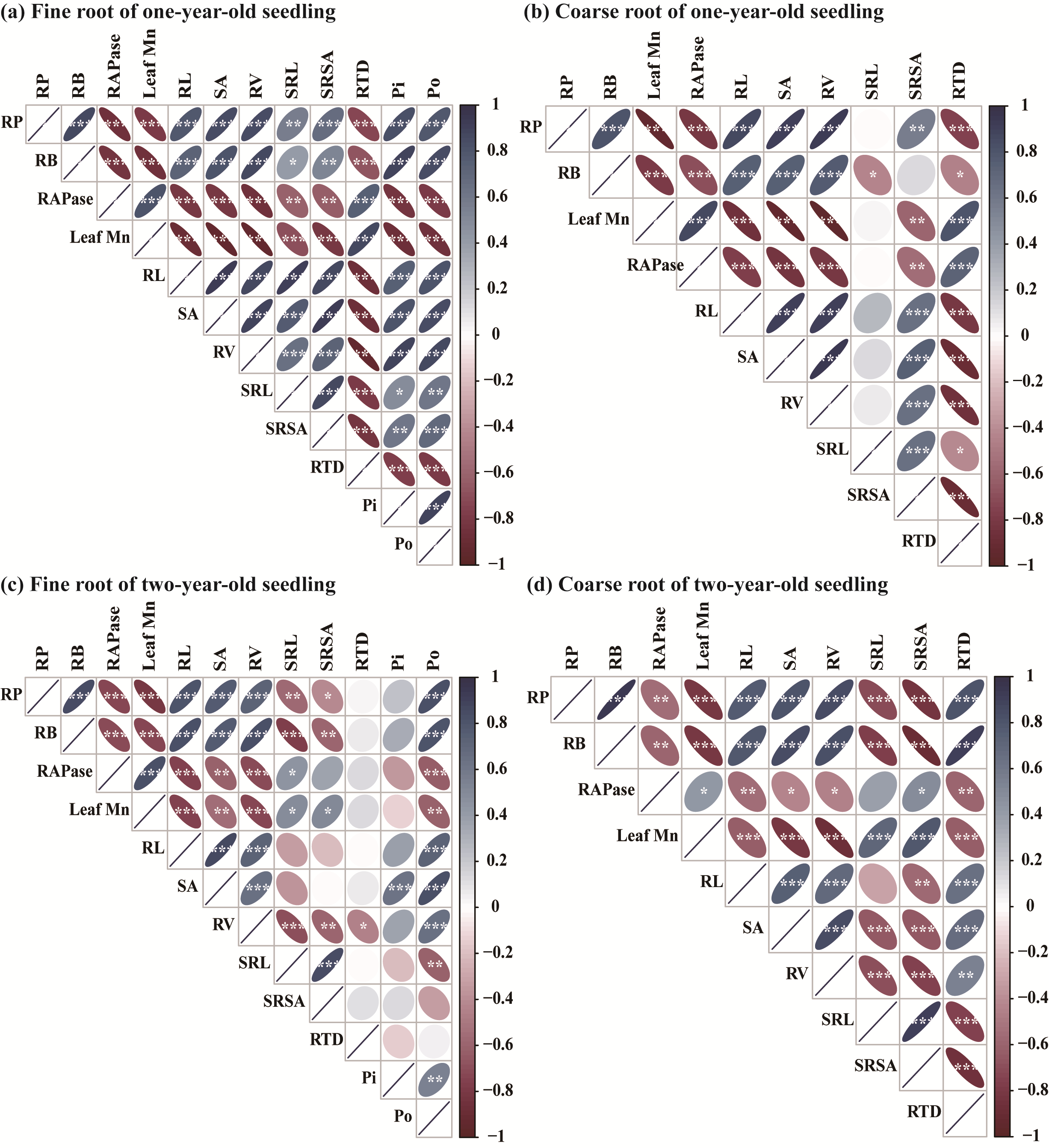


Note: Blue ellipse indicates positive correlation, and red ellipse indicates negative correlation. The shape and color depth of the ellipse represent the absolute value of the correlation, and the flatter the ellipse and the darker the color represent the greater the absolute value of the correlation. Asterisks represent the significance (* *P* < 0.05, ** *P* < 0.01, *** *P* < 0.001). RL: root length (cm); SRL: specific root length (cm mg^–1^); SA, root surface area (cm^2^); SRSA: specific root surface area (cm^2^ mg^–1^); RV, root volume (cm^3^); RTD: root tissue density (mg cm^–3^); FRB: fine root biomas (g plant^–1^);CRB: coarse root biomass (g plant^–1^); RAPase, root APase (μmol *p*NP g^−1^min^−1^); Leaf Mn (mg kg^–1^); RP, root phosphorus (g kg^–1^) ; Pi, soil inorganic P; and Po, soil organic P.
